# Encapsulation of *Lacticaseibacillus casei* and *Lactobacillus acidophilus* using *Elaeagnus angustifolia* L. flour as encapsulating material by emulsion method

**DOI:** 10.1002/fsn3.4328

**Published:** 2024-07-10

**Authors:** Büşra Karkar, Saliha Şahin, Lütfiye Yılmaz‐Ersan, Bekir Akça, Mesut Ertan Güneş, Cüneyt Özakın

**Affiliations:** ^1^ Faculty of Science and Arts, Department of Chemistry University of Bursa Uludağ Bursa Türkiye; ^2^ Faculty of Agriculture, Department of Food Engineering University of Bursa Uludağ Bursa Türkiye; ^3^ Faculty of Medicine, Department of Medical Microbiology University of Bursa Uludağ Bursa Türkiye; ^4^ Vocational School of Technical Sciences, Milk Technology Programme University of Bursa Uludağ Bursa Türkiye

**Keywords:** *Elaeagnus angustifolia* L., encapsulation, *Lacticaseibacillus casei*, *Lactobacillus acidophilus*, optimization, probiotic bacteria

## Abstract

In this study, *Lacticaseibacillus casei* and *Lactobacillus acidophilus* probiotic bacteria were encapsulated using oleaster flour, which is rich in phenolic compounds and has prebiotic properties as potential. The optimum conditions required for the encapsulation of *L*. *casei* and *L*. *acidophilus* bacteria with maximum efficiency using oleaster flour were determined by central composite design–response surface methodology. As a result of the optimization process, the encapsulation efficiency for *L*. *casei* and *L*. *acidophilus* capsules was 93.66 ± 2.58% and 74.97 ± 1.34%, respectively. The capsule sizes of *L*. *casei* and *L*. *acidophilus* encapsulated with oleaster flour were determined by scanning electron microscopy to be 104.8 ± 26.3 and 95.7 ± 12.1 μm, respectively. Fourier transform infrared spectroscopy analyses showed that there was no change in the structure of the encapsulation material, oleaster flour, after encapsulation. Also, the storage stability of free and encapsulated bacteria was investigated, and it was found that the viability losses of encapsulated probiotic bacteria were less than those of free probiotic bacteria. Finally, the effect of encapsulation on bacterial viability during in vitro gastrointestinal digestion was investigated, which is the main purpose of the study. While free probiotic bacteria cannot reach the intestinal environment alive after in vitro gastrointestinal digestion due to pH and enzyme effects, encapsulated *L*. *casei* and *L*. *acidophilus* bacteria largely preserved their viability, and their postdigestion viability was 39.59 ± 1.50% and 36.28 ± 0.01%, respectively. The results showed successful encapsulation of *L*. *casei* and *L*. *acidophilus* probiotic bacteria with oleaster flour.

## Graphical abstract

For the first time, *Lacticaseibacillus casei* and *Lactobacillus acidophilus* probiotic bacteria were encapsulated with oleaster (*Elaeagnus angustifolia* L.) flour. The encapsulation efficiency of *L*. *casei*–oleaster flour and *L*. *acidophilus*–oleaster flour microcapsules was found to be 93.66 ± 2.58% and 74.97 ± 1.34%, respectively. Compared to free probiotic bacteria, *L*. *casei*–oleaster flour and *L*. *acidophilus*–oleaster flour microcapsules were observed to preserve bacterial viability for a longer time under storage conditions. It has been determined that while free probiotic bacteria lose their viability during gastrointestinal digestion, encapsulated probiotic bacteria maintain their viability significantly.

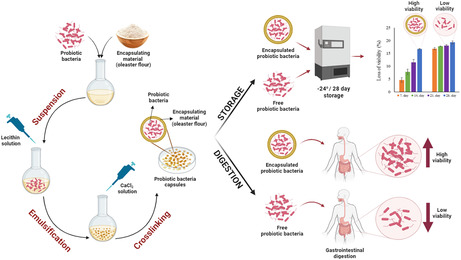

## 1 INTRODUCTION

Over the years, there has been an increasing interest in the connection between our food and health. This has led to functional foods, such as fortified dairy products and whole grains, which go beyond providing basic nutrition to offer additional health benefits. These functional foods help to prevent certain diseases and reduce the risk of developing them (Aboulfazli et al., 2015, 2016; Sengsaengthong & Oonsivilai, 2019). The approach adopted in recent years toward the creation and development of functional foods is the supplementation of probiotic bacterial strains.

Probiotics are live microorganisms, which, when administered in adequate amounts, confer health effects to the host (Hill et al., 2014). Composed of lactic acid bacteria like *Bifidobacterium* and *Lactobacillus*, these probiotics are a natural part of our intestinal microbiota (Açu et al., 2017; Homayouni et al., 2008; Niamah et al., 2018; Sengsaengthong & Oonsivilai, 2019). Their benefits are wide‐ranging, from preventing gastrointestinal infections to managing conditions like lactose intolerance, insulin resistance syndrome, Type 2 diabetes, and hypercholesterolemia (Galdeano et al., 2019; Kerry et al., 2018; Wan et al., 2019). They even possess therapeutic properties, acting as a shield against mutagenic, carcinogenic, diarrheal, and microbial threats (Galdeano et al., 2019; Kerry et al., 2018; Wan et al., 2019). In order to ensure that probiotic bacteria have a beneficial impact on health when ingested through tablets or food products, it is important to consider factors, such as their viability, stability, and compatibility with the product matrix. This includes the type of bacteria used, the daily dosage, the frequency and duration of consumption, and their ability to maintain the viability in the gastrointestinal (GI) tract (Boza‐Mendez et al., 2012; Santiago‐López et al., 2015). For probiotic bacteria to be effective in food products, they must survive all stages of food processing, storage, and the gastrointestinal tract and be able to compete with the intestinal microbiota. Various factors, such as the activity and dose level of probiotic bacteria, temperature, food product matrix, oxygen availability, pH value, and sugar concentration, can affect the viability of bacteria (Homayouni et al., 2008; Kataria et al., 2018). Encapsulation of probiotic cultures is one of the most efficient strategies recommended to increase the viability of probiotics by protecting them from adverse conditions (Akalin & Erişir, 2008; Champagne et al., 2015).

Encapsulation is a process that involves creating spherical particles of different sizes by encapsulating food ingredients, enzymes, and microorganisms with materials made of protein or carbohydrates. This step is crucial for storing probiotics for an extended period and releasing them in a controlled manner. Different methods for encapsulating probiotic bacteria include extrusion, emulsion, spray drying, and spray chilling (Cook et al., 2012; Uran et al., 2017). The emulsion method keeps probiotic bacteria in a water/oil or oil/water system by mixing polymer solutions like alginate, pectin, and carrageenan with vegetable oils, such as soy, corn, sunflower, and canola oil. After forming a water‐in‐oil (or oil‐in‐water) emulsion, the water‐soluble polymer should be insoluble in the oil phase to create capsules. After the emulsion, these capsules are hardened by adding a cross‐linker to the medium. Researchers have used various polymers, such as psyllium, xanthan gum, fenugreek, alginate, chitosan, and gum arabic, as encapsulating material when studying the encapsulation of probiotic bacteria (Uran et al., 2017). Using protein‐ and carbohydrate‐based materials with prebiotic properties as encapsulating material is also important for enhancing the viability of probiotic bacteria.

Oleaster is a plant from the *Elaeagnaceae* family that has medicinal properties and is commonly used in herbal medicine. It is an excellent source of essential fatty acids, vitamins, minerals, carotenoids, flavonoids, and other bioactive compounds (Ishaq et al., 2015; Khan et al., 2016; Okmen & Turkcan, 2014; Sabir et al., 2007; Saleh et al., 2018). Studies have shown that Oleaster has therapeutic effects, such as antioxidant, anti‐inflammatory, antimutagenic, antitussive, antitumor, anti‐arthritic, antimicrobial, and hepatoprotective properties (Incilay, 2014; Ishaq et al., 2015; Khan et al., 2016; Liao et al., 2012; Saleh et al., 2018). Oleaster flour, a natural product, is used in food products due to its unique taste, structure, dietary fiber, mineral, and phenolic components, as well as its prebiotic properties (Sabouri et al., 2019).

Nutrition plays a vital role in maintaining good health. Therefore, it is crucial to establish a clear link between nutrition and health safety. In recent times, people have become increasingly conscious of their health and dietary needs, which has led to a surge in demand for functional foods. To cater to this demand, researchers are exploring ways to create diverse functional food products. This study aimed to enhance the survival of *Lacticaseibacillus casei* and *Lactobacillus acidophilus* probiotic bacteria by encapsulating them with oleaster using the emulsion method. The study first determined the composition of oleaster flour in terms of total protein (TP), total fat (TF), total sugar (TS), total starch (TST), total carbohydrate (TC), and dietary fiber content. Then, the encapsulation conditions were optimized using a central composite design to achieve maximum encapsulation efficiency of *L*. *casei* and *L*. *acidophilus* bacteria by the emulsion method. The viability of *L*. *casei*–oleaster and *L*. *acidophilus*–oleaster capsules was compared to that of free *L*. *casei* and *L*. *acidophilus* bacteria during in vitro gastrointestinal digestion. The study also examined the viability rates of free and encapsulated *L*. *casei* and *L*. *acidophilus* bacteria during storage. Lastly, the antimicrobial properties of free and encapsulated *L*. *casei* and *L*. *acidophilus* bacteria were investigated using the well‐diffusion inhibition method.

## 2 MATERIALS AND METHODS

### 2.1 Preparation of encapsulating material

The oleaster flour (OLF) used in the encapsulation process was obtained from oleaster fruits collected from the Nevşehir province of Turkey. To make the OLF, the shell and seed parts of the oleaster fruits were separated and shredded. Then, the OLF was sifted through a 355‐mesh sieve to obtain a uniform texture. Finally, the OLF was stored at −24°C to maintain quality.

### 2.2 Chemical composition of OLF

The total protein (AOAC 991.20), total fat (AOAC 920.39), total sugar (AOAC 968.28), total starch (GMMAM), total carbohydrate (Cemeroğlu & Acar, 1986), dietary fiber (AOAC 991.43), moisture (TS EN ISO 712), ash (TS EN ISO 2171), dry matter and total titrable acidity (TS 4500, 2010) of OLF were determined using standard methods.

### 2.3 Sterilization of OLF

To avoid any negative effects due to contamination, the encapsulation material used in the encapsulation process must be sterilized. However, it is important to optimize the sterilization time to prevent any damage to the structure or chemical composition of the encapsulation material during the process. For this purpose, OLF was sterilized at 121°C for varying durations of 1, 2, 3, 4, and 5 min, in order to determine the optimum sterilization time. The total phenolic content, total fat, total sugar, total protein, total starch, and total carbohydrate contents of each sterilized sample were analyzed. The statistical analysis of the obtained data was performed using MINITAB 17.0 (Minitab Inc.) with one‐way analysis of variance (ANOVA) to determine the optimum sterilization time for OLF.

### 2.4 Activation and enhancement of probiotic bacterial strains

Two strains of probiotic bacteria, *Lacticaseibacillus casei* (DSM 20011) (LC) and *Lactobacillus acidophilus* (DSM 20079) (LA), were utilized in the study (Zheng et al., 2020). To activate the LC and LA bacterial strains, they were first placed in De Man, Rogosa, and Sharpe (MRS) broth (sterilized at 121°C for 15 min). The activation process was carried out by incubating the strains under anaerobic conditions at 37°C for 24 h, which was repeated thrice. The activated strains were then stored at −24°C until use. Before encapsulation, 1 mL of the stock probiotic bacterial culture was activated in 25 mL MRS medium by incubating at 37°C for 24 h (Ozcan et al., 2015).

### 2.5 Preparation of encapsulated probiotic bacterial strains

**FIGURE 1:**
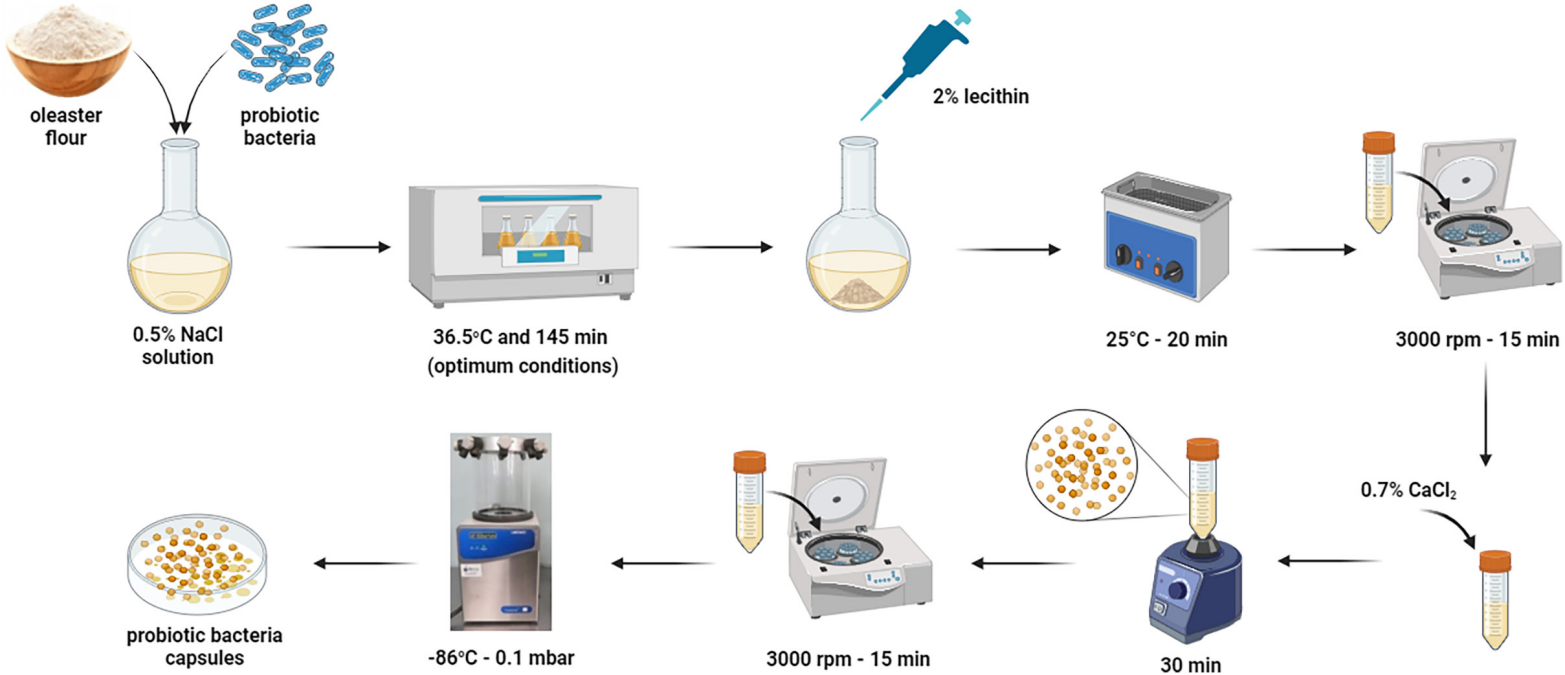
Encapsulation procedure of *Lacticaseibacillus casei* and *Lactobacillus acidophilus* probiotic bacteria with oleaster flour.

In this study, two types of lactic acid bacteria, LC and LA, were encapsulated using the emulsion technique (Homayouni et al., 2007). The encapsulating material used in the encapsulation process was OLF. An optimum amount of OLF was mixed with 50 mL of 0.5% sodium chloride (NaCl) solution and 25 mL of active probiotic bacteria to prepare the capsules. The mixture was then placed in a shaking incubator at the optimum temperature and time. Once it reached the optimum conditions, 2.5 mL of 2% lecithin was added to the mixture, and the emulsion process was carried out by keeping it in an ultrasonic bath at 25°C for 20 min. The sample was then centrifuged at 3000 rpm revolutions per minute (1050 g) for 15 min. Following this, 10 mL of 0.7% calcium chloride (CaCl_2_) was added to the residue, and the capsules were ripened by vortexing for 30 min. The mixture was centrifuged at 3000 rpm (1050 g) for 15 min, and the residue was lyophilized and stored at −24°C. The process of encapsulation is illustrated in Figure 1.

### 2.6 Optimization of encapsulation parameters of probiotic bacterial strains with OLF

The central composite design, which is one of the chemometric methods, was used to determine the optimum conditions for encapsulating probiotic bacteria on OLF (Şahin et al., 2024). The crucial factors were extraction temperature (°C), extraction time (min), and OLF amount (%). Table 1 provides the factors and coded level values used for the central composite design. Equation (1) was used to calculate the number of experiments (*N*) based on the number of factors (*k*) and repeated experiments (*x*_0_).

(1) $$N=2^{k}+2k+x_{0}$$

**TABLE 1:** Factors and level values for central composite design.

Variables	Levels				
	−1.18	−1	0	1	1.18
Extraction temperature (°C) (*x*_*1*_)	22	25	30	35	38
Extraction time (min.) (*x*_*2*_)	69.55	90	120	150	170.45
Amount of oleaster flour (%) (*x*_*3*_)	0.16	0.50	1.00	1.50	1.84

Where *k* is the number of factors and *x*_0_ is the number of repeated experiments. In this study, a five‐level, three‐factor central composite design was applied. The central composite design table given in Table 2 shows the parabolic effects of each factor. After carrying out 20 experiments, the encapsulated bacteria were released back into the phosphate buffer solution (pH 7–30 min) to determine the number of encapsulated bacteria. The encapsulation efficiency was then calculated, and ANOVA was performed using the Design‐Expert 7.0.0 software (Stat‐Ease, Inc.) program for the response values. Lastly, the quadratic polynomial equation (2) was used to determine the response variable, where y is the response variable, *b*_0_ is the constant term, *b*_*i*_ is the linear interaction constant, *b*_*ii*_ is the parabolic interaction constant, *b*_*ij*_ is the parameter interaction constant, and *x*_*i*_ and *x*_*j*_ are the independent variables.

(2) $$y=b_{0}+\sum_{i=1}^{3}b_{i}x_{i}+\sum_{i=1}^{3}b_{\mathrm{ii}}x_{i}^{2}+\sum_{i=1}^{2}\sum_{j=i+1}^{3}b_{\mathrm{ij}}x_{i}x_{j}$$

**TABLE 2:** The central composite design table.

Run order	Levels		
	*x*_*1*_	*x*_*2*_	*x*_*3*_
1	25.00	90.00	0.50
2	35.00	90.00	0.50
3	25.00	150.00	0.50
4	35.00	150.00	0.50
5	25.00	90.00	1.50
6	35.00	90.00	1.50
7	25.00	150.00	1.50
8	35.00	150.00	1.50
9	22.00	120.00	1.00
10	38.00	120.00	1.00
11	30.00	69.55	1.00
12	30.00	170.45	1.00
13	30.00	120.00	0.16
14	30.00	120.00	1.84
15	30.00	120.00	1.00
16	30.00	120.00	1.00
17	30.00	120.00	1.00
18	30.00	120.00	1.00
19	30.00	120.00	1.00
20	30.00	120.00	1.00

Abbreviations: *x*_1_, extraction temperature (°C); *x*_2_, extraction time (min.); *x*_3_, amount of oleaster flour (%).

### 2.7 Microbiological analysis

The LA and LC strains were counted using the pour‐plate technique on MRS‐Agar medium. The plates were incubated under anaerobic conditions at 37°C for 72 h. Activated‐free bacteria were transferred directly to serial dilutions, while encapsulated probiotic bacteria were added to serial dilutions after 30 min of mixing in phosphate buffer (pH 7). To prepare the plates, 1 mL of the serial dilutions was added to sterile plates. After adding 15–20 mL of MRS‐Agar medium, the plates were mixed by rotation and placed inverted in anaerogenic jars with AnaeroGen Gas Packs. The plates were left for 72 h of anaerobic incubation at 37°C. The colonies were counted on the plates and the results were reported as a log of colony‐forming units (cfu) per g‐encapsulated bacteria or mL‐free bacteria [log cfu/g (mL)] (Ozcan et al., 2010).

### 2.8 Morphology and size of probiotic bacteria capsules

The characterization of probiotic bacteria encapsulated with OLF was studied by Fourier transform infrared (FTIR) spectrometer (PerkinElmer, Spectrum 100). The sizes and surface morphology of probiotic bacteria encapsulated with OLF were studied by scanning electron microscopy (SEM) (Carl Zeiss Evo 40) at 10–20 kV. OLF–probiotic bacteria microcapsules were placed on aluminum plates and covered with gold.

### 2.9 Viability of free and encapsulated probiotic bacteria after in vitro gastrointestinal digestion

#### 2.9.1 Preparation of simulated gastric fluid and simulated intestinal fluid

To prepare simulated gastric fluid (SGF), 0.32 g of pepsin and 0.2 g of sodium chloride were added to 100 mL of water. The pH was adjusted to 2.0 ± 0.2 using 0.2 N hydrochloric acid (HCl) and 0.2 N sodium hydroxide (NaOH). The solution was sterilized with a disposable sterile filter (Tipigil, 2015).

To prepare simulated intestinal fluid (SIF), 7.7 mL of 0.2 N sodium hydroxide, 0.125 g of pancreatin, 0.3 g of bile salt, and 0.68 g of potassium dihydrogen phosphate were added to 100 mL of water. The pH was adjusted to 7.0 ± 0.2 using 0.2 N HCl and 0.2 N NaOH. The SIF was sterilized with a disposable sterile filter (Tipigil, 2015).

#### 2.9.2 Viability of free and encapsulated LA and LC after separate digestion in the gastrointestinal environment

To separately examine the digestion in the gastrointestinal environment, 9 mL of SGF and 9 mL of SIF medium were added to free probiotic bacteria (1 mL) and encapsulated probiotic bacteria (1 g) separately. The mixture was then incubated in a shaking incubator at 37°C for 0, 30, 60, 90, and 120 min. At the end of each digestion period, microbiological analyses of the samples were performed using peptone water and the survival rates of probiotic bacteria were recorded (Tipigil, 2015).

#### 2.9.3 Viability of free and encapsulated LA and LC after sequential digestion in the gastrointestinal environment

To examine sequential digestion in the gastrointestinal environment, free probiotic bacteria (1 mL) and encapsulated probiotic bacteria (1 g) were exposed to SGF (10 mL) for 90 min at 37°C in a shaking incubator. At the end of the digestion period, the pH of the medium was adjusted to 7.0 with 0.2 N NaOH and 10 mL of SIF was added. The mixture was then incubated in a shaking incubator at 37°C for 90 and 120 min. At the end of each digestion period, microbiological analyses of the samples were performed using peptone water and the survival rates of probiotic bacteria were recorded (Tipigil, 2015).

#### 2.9.4 Kinetic release patterns of probiotic bacteria capsules

The kinetic release profiles of LC and LA probiotic capsules in the in vitro gastrointestinal tract were studied using mathematical kinetic models. Various models, such as Zero‐order, First‐order, Higuchi, Hixson–Crowell, and Korsmeyer–Peppas models, were used to analyze the release profiles. The most appropriate kinetic release pattern was selected based on the highest correlation coefficient (*R* ^2^) obtained from the different equations applied (Dash et al., 2010; Mohammadi et al., 2023; Zahra et al., 2022).

### 2.10 Viability of probiotic bacteria during storage

Bacteria that were both encapsulated and free were stored at a temperature of −24°C for a period of 1 month. During this time, microbiological analyses were conducted on the samples on the 0th, 7th, 14th, 21st, and 28th days of storage. The cell population per g‐encapsulated bacteria or mL‐free bacteria [log cfu/g (mL)] was determined after the microbiological analysis, and the percentage loss of viability (%) was calculated over the storage period using Equation 3.

(3) $$\text{loss of viability}\;\left(\%\right)=\frac{\text{initial cell population}-\text{cell population}\;\mathrm{at}\;\text{storage time}\;}{\text{initial cell population}\;}\times 100$$

### 2.11 Antimicrobial analysis

The antimicrobial activity of free and encapsulated LC and LA probiotic bacteria was determined using the well‐diffusion inhibition method (Dagher et al., 2020). To evaluate antibacterial activity, the study selected the gram‐negative bacterial strain *Escherichia coli* (ATCC 25922) and the gram‐positive strains *Enterococcus faecalis* (ATCC 29212) and *Listeria monocytogenes* (ATCC 7644). Positive controls were used for each of these strains: *Trimethoprim–sulfamethoxazole* (SXT) for *E. coli*, *Vancomycin* (VA) for *E*. *faecalis*, and *Ampicillin* (AM) for *L*. *monocytogenes*. The Mueller–Hinton II Agar was used as the medium, and wells with an 8‐mm diameter were drilled. Before analysis, free probiotic bacteria were activated in MRS broth medium, and encapsulated probiotic bacteria were released in a pH 7 buffer. In each of the opened wells, bacterial cultures were added. The bacterial cultures included 180 μL of encapsulated LC, 180 μL of encapsulated LA, 180 μL of encapsulated LC + LA, 180 μL of free LC, 180 μL of free LA, and 180 μL of free LC + LA. After incubating at 37°C for 72 h under both anaerobic and aerobic conditions, the antibacterial activity was evaluated by measuring the diameter of the inhibition zone around each well.

## 3 RESULTS AND DISCUSSION

### 3.1 Chemical composition of OLF

The total protein, total fat, total sugar, total starch, total carbohydrate, dietary fiber, moisture, ash, dry matter, and total titrable acidity of OLF are given in Table 3. Cansev et al. (2011) found that the *Elaeagnus angustifolia* fruit's total protein content was 4.64 ± 0.88%; total soluble sugar content was 70.61 ± 3.76%; oil content was 0.47 ± 0.10%; moisture content was 26.52 ± 0.16%; ash content was 1.24 ± 0.09%; crude fiber content was 4.07 ± 0.19%, and total titratable acidity value was 1.84 ± 0.68%. When comparing the results with the findings of Cansev et al. (2011), it was generally observed that the OLF content was higher than that of oleaster fruit. This variation in the content can be attributed to factors, such as the region where the plant grows, the time of collection, the amount of light it receives, and the amount of nutrition and watering it is given.

**TABLE 3:** Chemical composition of OLF.

Chemical content (%)	
Total fat	11.67 ± 0.31
Total protein	5.98 ± 0.38
Total starch	10.70 ± 0.10
Total sugar	47.06 ± 3.38
Total carbohydrate	57.76 ± 3.38
Dry matter	71.35 ± 0.63
Moisture	28.65 ± 0.63
Ash	2.59 ± 0.02
Total titrable acidity	1.30 ± 0.01
Dietary fiber	19.65 ± 0.35

Abbreviation: OLF, oleaster flour.

### 3.2 Sterilization of OLF

The encapsulation process requires the coating material to be sterilized to prevent contamination‐induced loss of viability. The high temperatures required for sterilization must not cause any significant changes to the chemical structure of the OLF (coating material). Therefore, the sterilization time was optimized as determined in Section 2.3. The chemical content and statistical analysis results of OLF after sterilization are given in Table 4. Additionally, the chemical content of OLF before and after sterilization was statistically analyzed using ANOVA, and the similarity ratios were determined based on the *p*‐values obtained (Table 5). It has been observed that the physicochemical properties of unsterilized OLF and 1‐min sterilized OLF have a 24% similarity rate. However, this similarity rate decreases as the sterilization time increases. Therefore, the optimum sterilization time for OLF to be used in the encapsulation process has been determined as 1 min.

**TABLE 4:** Chemical content of oleaster flour before and after sterilization.

Time (min)	TF (%)	TP (%)	TST (%)	TS (%)	TC (%)	TPC (mg GAE/g)
–	11.67 ± 0.31	5.98 ± 0.38	10.70 ± 0.10	47.06 ± 3.38	57.76 ± 3.38	18.83 ± 1.09
1	12.31 ± 0.74	5.95 ± 0.38	3.90 ± 0.19	42.80 ± 3.07	46.70 ± 3.08	18.18 ± 0.83
2	12.39 ± 0.88	6.20 ± 0.40	10.12 ± 0.70	51.87 ± 3.73	61.99 ± 3.80	20.28 ± 0.41
3	14.29 ± 0.10	6.25 ± 0.40	6.54 ± 0.01	46.64 ± 3.35	53.18 ± 3.35	21.11 ± 0.51
4	11.71 ± 0.89	6.67 ± 0.43	16.08 ± 0.47	44.54 ± 3.20	60.62 ± 3.23	20.47 ± 0.50
5	11.82 ± 0.31	6.54 ± 0.42	18.70 ± 2.09	46.02 ± 3.31	64.73 ± 3.91	22.11 ± 0.46

Abbreviations: TC, total carbohydrate; TF, total fat; TP, total protein; TPC, total phenolic content; TS, total sugar; TST, total starch.

**TABLE 5:** Statistical analysis of oleaster flour sterilization.

	*p*‐Value
0–1 min	.76
0–2 min	.89
0–3 min	.96
0–4 min	.92
0–5 min	.82

### 3.3 Encapsulation of probiotic bacteria with OLF and determination of optimum conditions

The goal of the experiment was to determine the optimum conditions for encapsulating probiotic bacteria on OLF using the parameters outlined in Table 2. Microbiological analysis was conducted to determine encapsulation efficiency (%), and ANOVA of these efficiencies was performed using the Design‐Expert 7.0.0 software. After ANOVA, second‐order polynomial equations were determined for LC–OLF and LA–OLF capsules (Table 6).

**TABLE 6:** Second‐order polynomial equations for LC and LA.

Bacteria	Second‐order polynomial equations
LC	*y* = 82.62 + 2.79*x*_1_ − 0.074*x*_2_ − 1.13*x*_3_ − 0.59*x*_1_ *x*_2_ − 0.83*x*_1_ *x*_3_ + 1.98*x*_1_^2^ + 2.06*x*_2_^2^
LA	*y* = 62.60 − 1.73*x*_1_ − 0.68*x*_2_ − 0.81*x*_3_ + 1.04*x*_1_ *x*_2_ + 2.55*x*_2_ *x*_3_ + 1.64*x*_1_^2^ + 1.04*x*_2_^2^ + 2.90*x*_3_^2^

Abbreviations: LA, *Lactobacillus acidophilus*; LC, *Lacticaseibacillus casei*; *x*_1_, extraction temperature (°C); *x*_2_, extraction time (min); *x*_3_, amount of oleaster flour (%).

**FIGURE 2:**
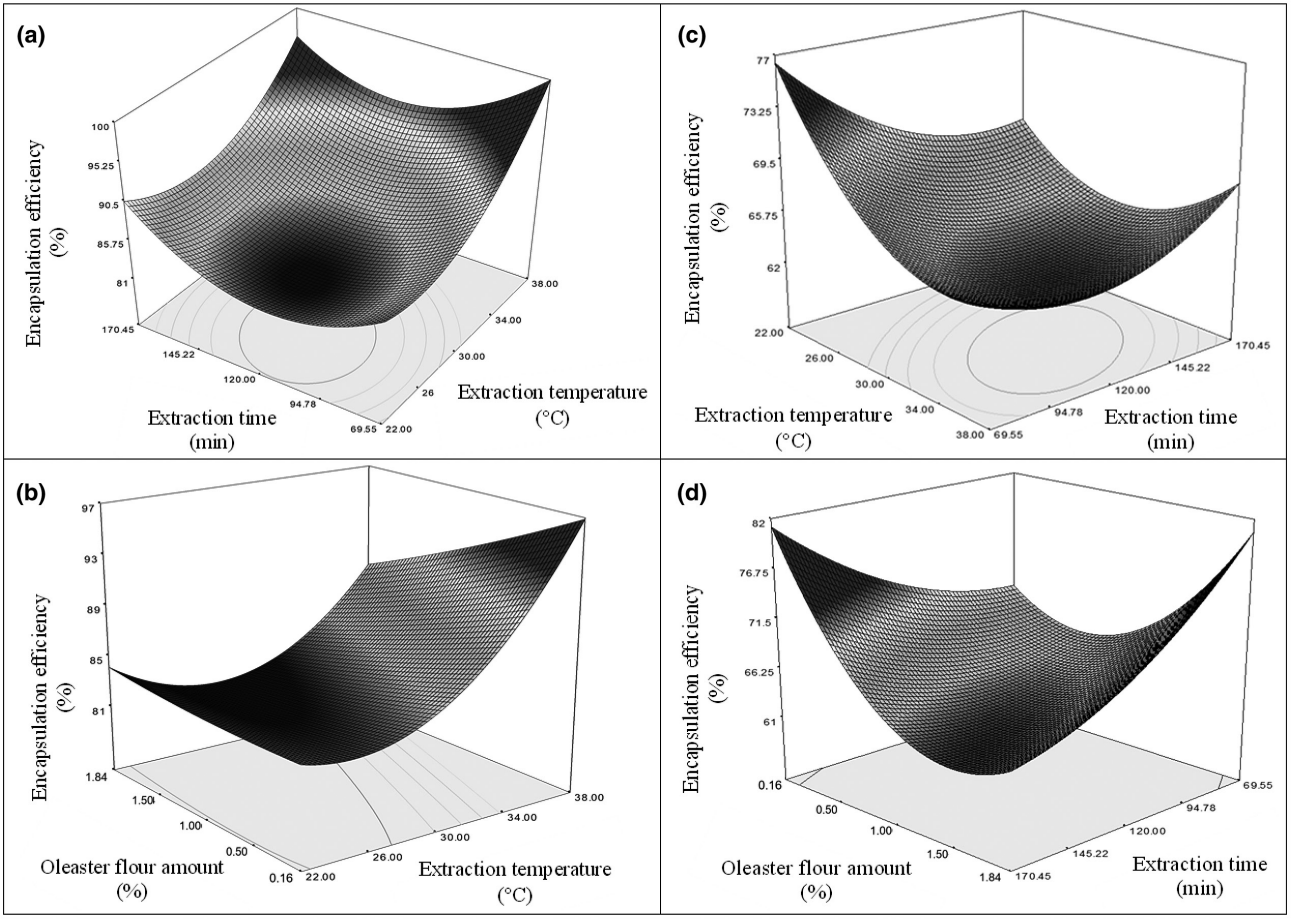
Three‐dimensional (3D) response surface analysis plots of encapsulation of probiotic bacteria with oleaster flour (OLF) (a) the effect of extraction time and extraction temperature on encapsulation efficiency of *Lacticaseibacillus casei* (LC) with OLF, (b) the effect of extraction temperature and oleaster flour amount on encapsulation efficiency of LC with OLF, (c) the effect of extraction time and extraction temperature on encapsulation efficiency of *Lactobacillus acidophilus* (LA) with OLF, and (d) the effect of extraction time and oleaster flour amount on encapsulation efficiency of LA with OLF.

Surface analysis graphs showing the bilateral interactions of factors effective in the encapsulation of LC and LA probiotic bacteria on OLF are given in Figure 2. While the extraction time was constant for the encapsulation efficiency of LC probiotic bacteria on OLF, the encapsulation efficiency was increased as the extraction temperature increased. While the extraction temperature was constant, as the extraction time was increased, the encapsulation efficiency decreased until a certain time but increased after a certain time. While the encapsulation efficiency was 100% when the extraction temperature was 38°C and the extraction time was 69 min, the yield decreased to 95% when the extraction time was increased to 170 min. When encapsulation was performed at 22°C for 170 min, the efficiency was around 90% (Figure 2a). When the effect of the amount of OLF and the extraction temperature was examined, it was seen that the encapsulation efficiency increased as the extraction temperature increased while the amount of OLF was constant. There was no meaningful change in the encapsulation efficiency as the amount of OLF increased while the extraction temperature was constant. While the amount of OLF was 0.16%, the encapsulation efficiency reached 97% when the encapsulation process was conducted at a high temperature (38°C) (Figure 2b).

While the extraction time was constant for the encapsulation efficiency of LA probiotic bacteria on OLF, the encapsulation efficiency decreased up to a certain temperature (34°C) as the extraction temperature increased, and the encapsulation efficiency started to increase as the temperature continued to increase. When the encapsulation process was conducted at 22°C and for 69 min, the encapsulation efficiency reached the maximum level (77%) (Figure 2c). When the effect of the amount of OLF and the extraction time was examined, while the amount of OLF was constant, the encapsulation efficiency decreased as the extraction time increased. When the extraction time was kept constant, the encapsulation efficiency decreased until the amount of OLF increased from 0.16% to around 1.00%, and the encapsulation efficiency increased when the amount of OLF was increased more. It was seen that the encapsulation efficiency was highest at the lowest extraction time when the maximum amount of oleaster flour was used (Figure 2d).

Table 7 presents the analysis of variance (ANOVA) for the quadratic model of the response surface of the encapsulation efficiency of probiotic bacteria. Table 8 shows the experimental and predicted values (encapsulation yield, %) for the yield percentage obtained from encapsulation. Optimum conditions for extraction temperature, time, and OLF amount were determined through ANOVAs using the Design‐Expert 7.0.0 software (Stat‐Ease, Inc.) program. The optimum conditions for maximum encapsulation of LC and LA bacteria with OLF were determined as follows: the extraction temperature was 36.5°C for both bacteria, the extraction times were 142.47 min for LC and 145.41 min for LA, and the amounts of OLF used were 0.60% for LC and 0.68% for LA (Table 9). Table 9 presents the predicted and experimental values (encapsulation yield, %) under these optimum conditions. The encapsulation efficiency was found to be 93.66 ± 2.58% for LC–OLF probiotic bacteria capsule and 74.97 ± 1.34% for LA–OLF capsule.

**TABLE 7:** Analysis of variance (ANOVA) for response surface quadratic model.

Source	*Lacticaseibacillus casei* (*R* ^2^ = .9888)					*Lactobacillus acidophilus* (*R* ^2^ = .9511)				
	DF	SS	MS	*F* value	*p* Value	DF	SS	MS	*F* value	*p* Value
Model	9	240.81	26.76	97.88	<.0001	9	271.69	30.19	21.62	<.0001
Lack of fit	5	2.28	0.46	5.02	.0505	5	9.59	1.92	2.19	.2049
Pure error	5	0.45	0.091			5	4.38	0.88		

Abbreviations: DF, degree of freedom; MS, mean square; SS, sum of squares.

**TABLE 8:** The predicted and experimental response values for the encapsulation of LC and LA probiotic bacteria.

Run order	Levels			LC (encapsulation yield, %)		LA (encapsulation yield, %)	
	*x*_*1*_	*x*_*2*_	*x*_*3*_	Experimental	Predicted	Experimental	Predicted
1	−1	−1	−1	83.40	83.79	70.90	69.52
2	1	−1	−1	92.50	92.19	64.45	64.73
3	−1	1	−1	85.42	85.39	70.74	71.18
4	1	1	−1	91.40	91.45	71.68	70.55
5	−1	−1	1	83.40	83.76	73.52	73.74
6	1	−1	1	88.42	88.86	68.80	67.45
7	−1	1	1	83.48	84.21	66.40	65.20
8	1	1	1	86.93	86.96	62.62	63.08
9	−1.18	0	0	84.50	83.84	69.45	70.15
10	1.18	0	0	93.14	93.22	63.75	64.34
11	0	−1.18	0	89.20	88.88	65.79	66.67
12	0	1.18	0	88.89	88.63	63.98	64.39
13	0	0	−1.18	85.00	85.14	71.56	72.18
14	0	0	1.18	82.07	81.34	68.77	69.44
15	0	0	0	83.08	82.92	62.26	62.60
16	0	0	0	83.16	82.92	61.27	62.60
17	0	0	0	82.39	82.92	64.00	62.60
18	0	0	0	83.17	82.92	62.23	62.60
19	0	0	0	82.82	82.92	63.16	62.60
20	0	0	0	82.78	82.92	62.90	62.60

Abbreviations: LA, *Lactobacillus acidophilus*; LC, *Lacticaseibacillus casei*; *x*_1_, extraction temperature (°C); *x*_2_, extraction time (min); *x*_3_, amount of oleaster flour (%).

**TABLE 9:** The responses of predicted and experimental values and their optimum conditions.

Responses	Optimum conditions			Encapsulation yield (%)	
	*x*_*1*_	*x*_*2*_	*x*_*3*_	Predicted	Experimental
LC	36.50	142.47	0.60	93.84	93.66 ± 2.58
LA	36.50	145.41	0.68	74.00	74.97 ± 1.34

Abbreviations: LA, *Lactobacillus acidophilus*; LC, *Lacticaseibacillus casei*; *x*_1_, extraction temperature (°C); *x*_2_, extraction time (min); *x*_3_, amount of oleaster flour (%).

It has been suggested that LC and LA probiotic bacteria, which produce lactic acid due to fermentation, can be used as additives in food products, particularly dairy products, to enhance their functional properties. LC and LA probiotic bacteria were encapsulated with skimmed milk powder to test this hypothesis. During encapsulation, skimmed milk powder with 0.2% fat, 33.0% protein, and 54.1% lactose content was used as an encapsulating material. The LC–skimmed milk powder capsules had an encapsulation efficiency of 86.43 ± 1.66%, which was lower than that of LC–OLF capsules (93.66 ± 2.58). The encapsulation efficiency of LA–skimmed milk powder capsules was 76.54 ± 2.15%, while the encapsulation efficiency of LA–OLF capsules was approximately 1.57% lower (74.97 ± 1.34%). These results indicate that OLF can be used as an alternative to skimmed milk powder to encapsulate probiotic bacteria, resulting in higher‐yield capsules with improved viability.

### 3.4 FTIR analysis of probiotic bacteria capsules

**FIGURE 3:**
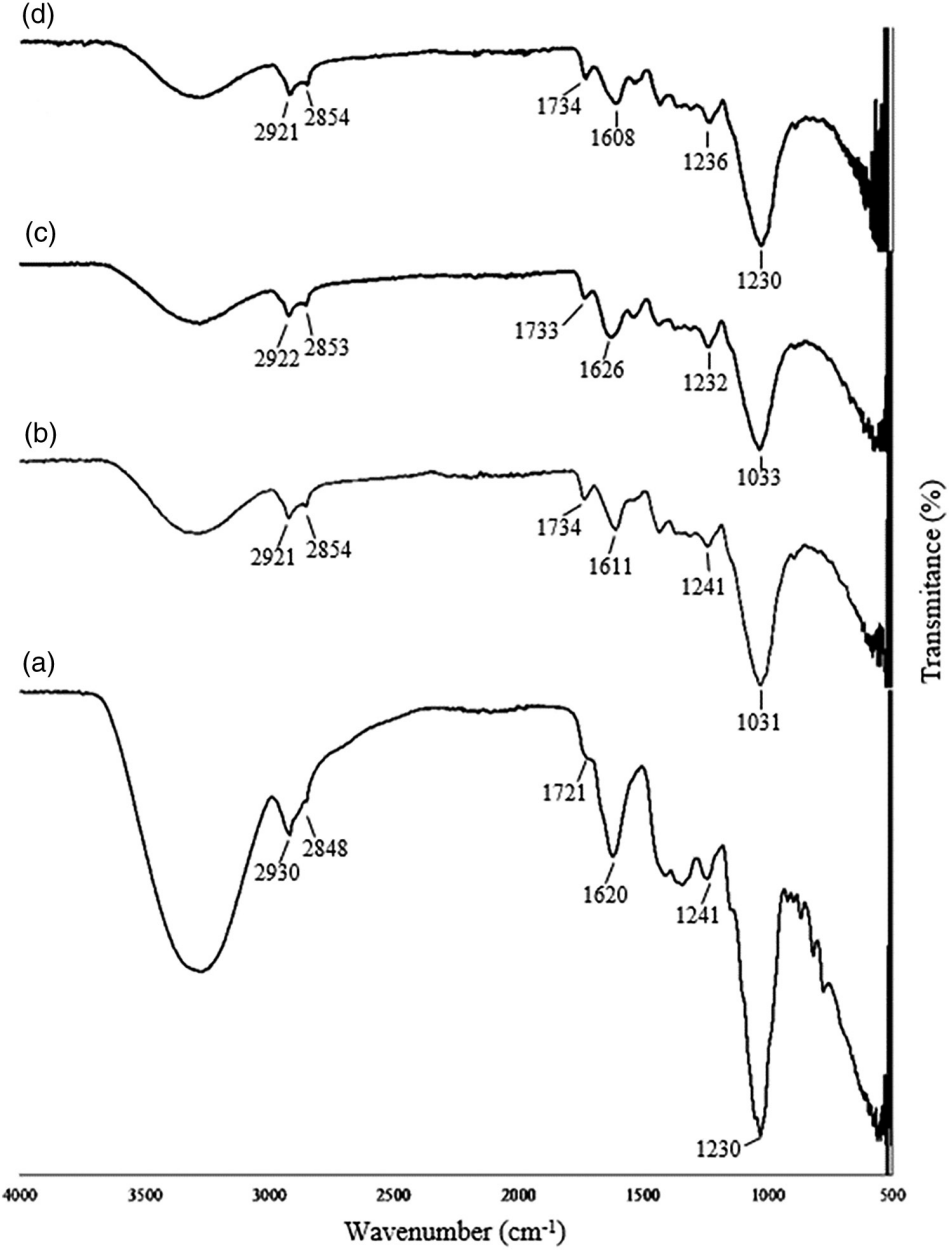
The Fourier transform infrared spectra of different oleaster flour (OLF) samples (a) OLF alone, (b) encapsulated OLF without probiotic bacteria, (c) LC–OLF capsules, and (d) LA–OLF capsules.

Fourier transform infrared (FTIR) spectra were used as a complementary technique for the postencapsulation structure analysis of OLF samples. The results of FTIR analysis for OLF sterilized at 121°C for 1 min, and for OLF in which the bacteria‐free encapsulation procedure was used, as well as for LC–OLF and LA–OLF capsules are presented in Figure 3. The absorption band in the wavenumber range of 3600–3000 cm^−1^ represents the stretching vibrations of –OH groups resulting from the presence of hydrogen bonds. It is seen that the hydrogen bond density decreases with the encapsulation process. Bands in the 3000–2800 cm^−1^ range represent molecular and aromatic C–H (El Mouftari et al., 2021; Hirri et al., 2015). Bands in the range of 2946–2881 cm^−1^ represent C–H (CH_2_) asymmetric stretching vibrations, bands in the range of 2881–2782 cm^−1^ C–H (CH_2_) symmetric stretching vibrations, bands in the range of 1486–1446 cm^−1^ C–H (CH_2_) bending (shear) vibrations, and bands in the range of 1382–1371 cm^−1^ C–H (CH_2_) bending (symmetric) vibrations. Bands in the range of 1290–1072 cm^−1^ indicate the presence of C–O (alcohol) stretching vibrations. Bands in the range of 1795–1677 cm^−1^ represent C–O (ester) stretching vibrations, indicating the presence of triglyceride and functional group carbonyl ester of triglycerides (El Mouftari et al., 2021; Guillén & Cabo, 1997; Rohman & Man, 2010). Bands in the 1200–1000 cm^−1^ range are due to stretching vibrations in the pyranose ring in monosaccharides (C=O, C–O–C, and C–OH). Bands in the 1000–800 cm^−1^ range indicate glycosidic bonds (Vaziri et al., 2018). In addition, the spectral region in the range of 1500–500 cm^−1^ represents the C–H stretching and stretching vibrations of hydrocarbons and the aromatic C=C–C vibrations (El Mouftari et al., 2021).

Upon examining the spectra, no significant difference was observed in the structure of the OLF before and after encapsulation. It can be concluded that encapsulation did not cause any deterioration in the structure of the OLF. The fact that the encapsulating material does not change during the encapsulation process is particularly important for the optimum encapsulation of probiotic bacteria and preserving their viability. It is thought that there is an increase in the carboxyl group because of cross‐linking with Ca^2+^. The vibrations of the C–O bond in the carboxyl group were observed at the b, c, and d spectra in 1734, 1733, and 1734 cm^−1^ wave numbers, respectively (Figure 3). In addition, it is thought that the small absorption changes observed at 1550 cm^−1^ wave number may result from electrostatic interactions with amino groups. It shows that the slight changes observed in the FTIR spectrum are due to hydrogen and van der Waals bonds that occur during the encapsulation of probiotic bacteria into the OLF.

### 3.5 Surface analysis and determination of the size of probiotic bacteria capsules

**FIGURE 4:**
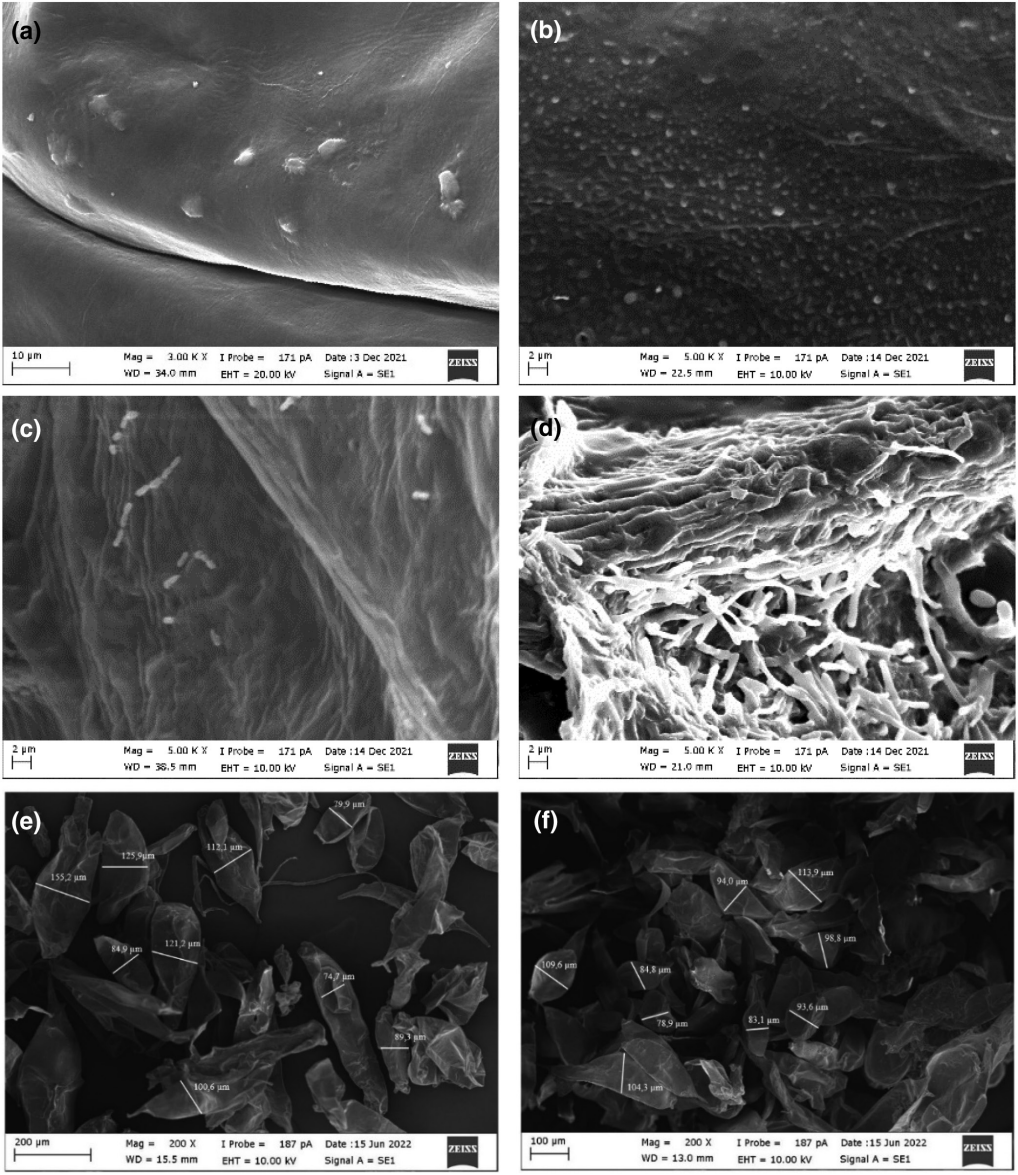
The SEM images of different oleaster flour (OLF) samples (a) OLF alone, (b) encapsulated OLF without probiotic bacteria, (c) LC–OLF capsules, (d) LA–OLF capsules, (e) dimensions of LC–OLF capsules, and (f) dimensions of LA–OLF capsules.

Scanning electron microscopy (SEM) image of the OLF sample, which was sterilized at 121°C for 1 min, was taken with 3000× magnification (Figure 4a). In the taken SEM image, it is seen that the sterilized OLF surface is quite smooth. The SEM images were taken with 5000× magnification of the OLF sample, in which the encapsulation procedure was applied without bacteria (Figure 4b). When the surface was examined, it was observed that wet spheres were formed on the surface of the OLF due to the cross‐links between the carboxylate and Ca^2+^ ions (Liu et al., 2004; Vaziri et al., 2018). The SEM images were taken with 5000× magnification after lyophilization of the OLF samples, in which LC probiotic bacteria were encapsulated under optimum conditions (Figure 4c). It has been observed that probiotic bacteria form clusters in the formed LC–OLF capsules and are mostly embedded in the OLF in the form of sticks; an exceedingly small part of them clings to the surface, and the surfaces become rough. The SEM images were taken with 5000× magnification after lyophilization of the OLF samples, in which LA probiotic bacteria were encapsulated under optimum conditions (Figure 4d). It has been observed that probiotic bacteria form clusters in the capsules of LA–OLF and are embedded in the OLF in the form of sticks, and the surfaces become rough, just like in the capsules of LC–OLF. It was concluded that all the bacteria were encapsulated inside the capsules since probiotic bacteria were rarely seen on the surface. The sphericity of LC–OLF and LA–OLF capsules deteriorated after lyophilization and turned into a rough surface with an irregular and less spherical structure. The average dimensions of the LC–OLF capsules were found to be 104.8 ± 26.3 μm (Figure 4e), while the average size of the LA–OLF capsules was determined as 95.7 ± 12.1 μm (Figure 4f).

### 3.6 Survival of free and encapsulated probiotic bacteria during exposure to gastrointestinal conditions

Probiotic bacteria taken with food products undergo the first cellular stress in the gastric medium after they are taken into the body. It has been proven in many studies that bacteria lose their viability after consumption and cannot reach the gastrointestinal system in sufficient quantities. For this reason, it is important to cover the bacteria with the encapsulation technique and protect them with a physical barrier against environmental conditions (Gül, 2015). The viability of free probiotic bacteria, LC–OLF and LA–OLF probiotic bacteria capsules was investigated in the gastric, intestine, and sequential gastrointestinal environments for 120 min (Table 10). This study observed that encapsulated probiotic bacteria significantly preserved their viability compared to free bacteria in gastrointestinal conditions. The viabilities of free LA and LC probiotic bacteria exposed to digestion in SGF medium for 120 min were observed to be completely inactive within 30 and 60 min, respectively. Similarly, the LA–OLF and LC–OLF capsules underwent digestion in SGF medium for 120 min. After digestion, bacterial viabilities were measured at 3.00 log cfu/g and 5.06 log cfu/g, respectively (Table 10). It was observed that there was no meaningful change in the viability of free and encapsulated probiotic bacteria mixed in a SIF medium for 120 min (Table 10). In many studies, the time determined for digestion in the SGF differs between 90 and 120 min (Gül, 2015). In the present study, the mixing time of probiotic bacteria in the SGF was determined to be 90 min in sequential gastric and intestinal environment studies, since there was not much change in the number of live microorganisms between 90 and 120 min. Free and encapsulated bacteria mixed in an SGF medium for 90 min were added to the SIF medium directly after the pH of the medium was adjusted to 7 and mixed for 90–120 min. Since free bacteria could not survive in the SGF environment for 90 min, they were not shown viability in the sequential gastrointestinal environment. The viability rates for LC–OLF and LA–OLF, mixed in the SIF for 90 min after the SGF, were 54.40% and 45.05%, respectively. The viability rates for LC‐OLF and LA‐OLF, mixed in the SIF for 120 min after the SGF, were 39.59% and 36.28%, respectively (Table 10). While probiotic bacteria taken into the body in free form could not reach the intestinal environment, it was observed that the probiotic bacteria encapsulated on OLF reached the intestinal environment and could maintain their viability.

**TABLE 10:** Survival of free and encapsulated probiotic bacteria during exposure to gastrointestinal conditions.

	SGJ			SIJ			SGJ–SIJ		
	Time (min)	Number of viable probiotic bacteria*	Viability (%)	Time (min)	Number of viable probiotic bacteria*	Viability (%)	Time (min)	Number of viable probiotic bacteria*	Viability (%)
LCF	0	7.03 ± 0.02	97.49 ± 0.29	0	7.25 ± 0.01	100.5 ± 0.12	90	nd.	0
	30	3.93 ± 0.04	54.49 ± 0.50	30	7.20 ± 0.03	99.84 ± 0.37			
	60	nd.	0	60	7.10 ± 0.5	98.51 ± 0.69			
	90	nd.	0	90	6.91 ± 0.01	95.81 ± 0.15	120	nd.	0
	120	nd.	0	120	6.80 ± 0.02	94.28 ± 0.27			
LCE	0	9.02 ± 0.13	93.92 ± 1.38	0	9.59 ± 0.17	99.86 ± 1.78	90	5.22 ± 0.76	54.40 ± 7.87
	30	7.00 ± 0.02	72.95 ± 0.18	30	8.81 ± 0.18	91.73 ± 1.84			
	60	5.56 ± 0.04	57.87 ± 0.45	60	8.63 ± 0.51	89.90 ± 5.30			
	90	5.17 ± 0.04	53.86 ± 0.44	90	8.38 ± 0.63	87.25 ± 6.55	120	5.22 ± 0.14	39.59 ± 1.50
	120	5.06 ± 0.17	52.74 ± 1.76	120	8.14 ± 0.85	84.84 ± 8.88			
LAF	0	7.75 ± 0.27	96.70 ± 3.38	0	7.98 ± 0.03	99.68 ± 0.35	90	nd.	0
	30	nd.	0	30	7.85 ± 0.02	98.05 ± 0.30			
	60	nd.	0	60	7.66 ± 0.09	95.63 ± 1.13			
	90	nd.	0	90	7.20 ± 0.07	89.94 ± 0.82	120	nd.	0
	120	nd.	0	120	6.79 ± 0.24	84.72 ± 2.98			
LAE	0	7.88 ± 0.09	95.23 ± 1.06	0	8.04 ± 0.03	97.19 ± 0.39	90	3.73 ± 0.11	45.05 ± 1.29
	30	6.21 ± 0.02	75.06 ± 0.26	30	7.83 ± 0.01	94.65 ± 0.09			
	60	3.54 ± 0.09	42.80 ± 1.07	60	7.69 ± 0.23	92.98 ± 2.81			
	90	3.39 ± 0.12	40.98 ± 1.51	90	7.18 ± 0.16	86.87 ± 1.89	120	3.00 ± 0.01	36.28 ± 0.01
	120	3.00 ± 0.01	36.28 ± 0.01	120	6.92 ± 0.38	83.62 ± 4.59			

*Note*: Mean ± standard deviation.

Abbreviations: LAE, encapsulated *Lactobacillus acidophilus*; LAF, free *Lactobacillus acidophilus*; LCE, encapsulated *L*. *casei*; LCF, free *L*. *casei*; nd, not detected; SGJ, simulated gastric juice; SIJ, simulated intestinal juice.

* log cfu/g (for encapsulated probiotic bacteria), log cfu/mL (for free probiotic bacteria).

When the literature was examined, *Lactobacillus plantarum* probiotic bacteria were encapsulated with alginate using extrusion and electrospray methods, and the viability of the obtained microcapsules in SGF–SIF media was examined. While the initial viable bacterial count of the control sample was 7.5 ± 0.20 log cfu/mL, there was a loss of viability of 3.3 log units after exposure to SGF–SIF media. While the initial viable bacterial count of the microcapsules obtained by the extrusion method was 7.42 ± 1.21 log cfu/mL, a 0.52 log unit decrease was observed in viability after exposure to SGF–SIF media. While the initial viable cell count of the microcapsules obtained by the electrospray method was 7.16 ± 1.05 log cfu/mL, there was a loss of viability of 1.11 log units after exposure to SGF–SIF environments. The results showed that *L*. *plantarum* probiotic bacteria encapsulated by extrusion and electrospray methods were much more resistant to SGF–SIF media than free probiotic bacteria (Tipigil, 2015). In another study, *L*. *casei* Shirota probiotic bacteria were encapsulated in alginate by extrusion and emulsion methods. They were kept in the SGF environment for 90 min and then in the SIF environment for 90 min, and their viability was determined. While the viability losses of the capsules obtained by extrusion and emulsion methods in the gastric environment were found to be between 2.08–2.83 and 2.62–3.21 log cfu/g, respectively, free *L*. *casei* Shirota probiotic bacteria completely lost their viability. There was no significant change in the viability rates of the capsules taken into the intestinal environment afterwards (Gül, 2015).

Similarly, in the in vitro gastrointestinal study conducted with free and encapsulated *Bifidobacterium adolescentis* bacteria, it was observed that there was a loss of viability in the first 60 min of the gastric environment. However, the viability level in the intestinal environment did not change (Annan et al., 2008). A study conducted with three different strains of *Lactobacillus* determined that free bacteria in the simulated gastric environment lost their viability in the 90th min (Gbassi et al., 2009).

The in vitro gastrointestinal release data of LC–OLF and LA–OLF capsules were subjected to various mathematical models to predict the release kinetics and mechanisms of the probiotic bacteria. Table 11 presents the values of the regression coefficients (*R* ^2^) of LC–OLF and LA–OLF capsules separately and sequentially for each model after gastrointestinal digestion. The most appropriate method was determined based on the regression coefficient. The ‘*R* ^2^’ values for zero order were found to be larger than those of the other models, indicating conformity to the zero‐order release kinetic pattern of digestion of LC–OLF and LA–OLF capsules separately in the gastric and intestinal environments. According to this kinetic model, the release of probiotic bacteria from capsules in gastric and intestinal environments is controlled by pore diffusion and is not affected by the amount of viable bacteria. The sequential gastric and intestinal digestion release pattern of LC–OLF and LA–OLF capsules conforms to the first‐order release kinetic pattern. First‐order release kinetics suggests that the change in bacterial viability over time depends on the number of viable bacteria (Dash et al., 2010; Mohammadi et al., 2023; Zahra et al., 2022). These findings underscore the power of mathematical models in understanding the release kinetics and mechanisms of probiotic bacteria, providing valuable insights for future research and development in the field.

**TABLE 11:** Gastrointestinal release patterns of encapsulated LC and LA probiotic bacteria.

Medium	Model	*R* ^2^	
		LC capsules	LA capsules
SGF	Zero‐order	**.9906**	**.9808**
	First‐order	.9862	.9749
	Higuchi	.8548	.8238
	Hixson–Crowell	.9878	.9770
	Korsmeyer–Peppas	.9078	.8435
SIF	Zero‐order	**.9854**	**.9492**
	First‐order	.9838	.9472
	Higuchi	.8438	.7660
	Hixson–Crowell	.9844	.9479
	Korsmeyer–Peppas	.5442	.4425
SGF + SIF	Zero‐order	.9711	.9668
	First‐order	**.9745**	**.9728**
	Higuchi	.9171	.9131
	Hixson–Crowell	.9738	.9712
	Korsmeyer–Peppas	.9264	.8837

Abbreviations: LA, *Lactobacillus acidophilus*; LC, *Lacticaseibacillus casei*; SGF, simulated gastric fluid; SIF, simulated intestinal fluid.

The most appropriate R2 values were shown as bold.

### 3.7 Viability of probiotic bacteria during storage

**FIGURE 5:**
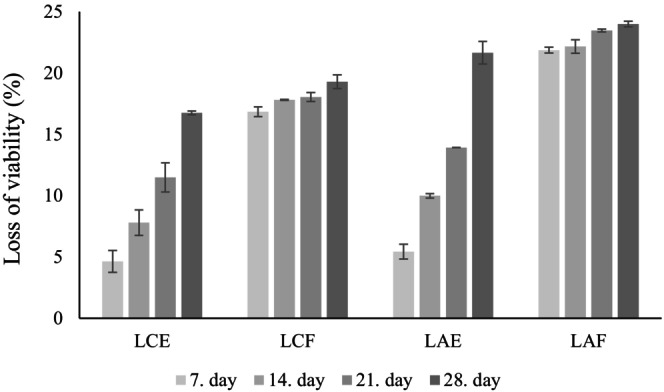
Loss of viability (%) of probiotic bacteria during storage (LAE, encapsulated *L*. *acidophilus*; LAF, free *L*. *acidophilus*; LCE, encapsulated *L*. *casei*; LCF, free *L*. *casei*).

Encapsulated and free probiotic bacteria were stored at −24°C for 28 days. To evaluate bacterial viability, microbiological analysis was performed weekly, and viability losses are given in Figure 5. The results showed that while the viability of encapsulated probiotic bacteria gradually decreased over a month, the free probiotic bacteria experienced a sudden decrease on the 7th day, and no increase or decrease was observed for 1 month. After 7 and 28 days of storage, the viability loss observed in LC–OLF capsules was 4.64% and 16.75%, respectively. On the other hand, the free LC bacteria had a loss of viability of 16.84% and 19.29% after 7 and 24 days of storage, respectively. Similarly, the loss of viability in LA‐OLF capsules and free LA bacteria was 5.43% and 21.87%, respectively, on the 7th day of storage. On the 28th day of storage, the loss of viability was found to be 21.66% and 23.99%, respectively. Overall, the loss of viability after 1 month of storage in LC probiotic bacteria was below 20% and below 25% in LA probiotic bacteria. Encapsulation provided a barrier against the environment during storage, which made the encapsulated probiotic bacteria even more resistant to environmental conditions than free probiotic bacteria. Moreover, OLF, which has prebiotic properties, acted as a food source for the bacteria, and encapsulated probiotic bacteria preserved their viability better than free probiotic bacteria (Sabouri et al., 2019). To summarize, encapsulation of probiotic bacteria with OLF effectively maintains their viability during storage.

In research, *L*. *acidophilus* KPb4b bacteria were encapsulated using different alginate concentrations with emulsion method and stored at +5°C for 4 weeks. With the increase in alginate concentration, it was observed that more bacteria remained alive during storage (İşleyen, 2018). In another research, *L*. *casei* Shirota probiotic bacteria were encapsulated with the emulsion method by using alginate, starch, gelatin, and chitosan at different rates. The capsules were stored at 4 and 24°C for 8 weeks, and their viability was examined. Free bacteria completely lost their viability in the 4th week of storage. The viability loss of the bacteria in the capsules obtained by the emulsion method was observed to be between 16.97% and 31.48% (Gül, 2015).

### 3.8 Antimicrobial analysis

The antimicrobial effects of free and encapsulated LC and LA probiotic bacteria against *E*. *coli*, *E*. *faecalis*, and *L*. *monocytogenes* bacteria were investigated under aerobic and anaerobic conditions (Table 12). After 72 h of incubation under aerobic conditions, zone of inhibition was not grown in the disks of positive control, free, and encapsulated probiotic bacteria. Therefore, it was concluded that LC and LA probiotic bacteria did not show antibacterial activity against *E*. *coli*, *E*. *faecalis*, and *L*. *monocytogenes* bacteria under aerobic conditions. The antimicrobial activity against *E*. *faecalis* was not observed after 72 h of incubation under anaerobic conditions. An inhibition zone of 18 mm in diameter was grown in the positive control (AM) disk against *L*. *monocytogenes*, while inhibition was not observed in free and encapsulated probiotic bacteria. It has been demonstrated that LC and LA do not possess antimicrobial properties against *L*. *monocytogenes*. In the test, a 30‐mm inhibition zone was formed on the positive control (SXT) disk against *E*. *coli* under anaerobic conditions. No inhibition zone was observed in the encapsulated LA, free or encapsulated LC, and free or encapsulated LC + LA probiotic bacterial disks. However, a 12‐mm inhibition zone was seen in the disk where free LA probiotic bacteria were embedded. The presence of an inhibition zone indicates antimicrobial activity. It was determined that free LA exhibited 40% antimicrobial activity against *E*. *coli* bacteria compared to the positive control. The encapsulated bacteria were found to not exhibit antimicrobial activity. In a study by Shim et al. (2016), antimicrobial activity against *E*. *coli* was investigated using six different *Lactobacillus* strains. The results revealed that the bacterial strains exhibited moderate antimicrobial activity, with zone diameters ranging from 10.5 to 20.5 mm. Furthermore, other studies involving *Lactobacillus* strains have shown varied results, ranging from absence of inhibition to high inhibition (Shim et al., 2016).

**TABLE 12:** Antimicrobial activity of free and encapsulated LC and LA probiotic bacteria.

Anaerobic conditions	Diameter of growth inhibition zone (including well diameter), mm^a^						
		LA	LC		LA + LC		
	Positive control	Free	Encapsulated	Free	Encapsulated	Free	Encapsulated
*Escherichia coli*	30	12	no inh.	no inh.	no inh.	no inh.	no inh.
*Enterococcus faecalis*	no inh.	no inh.	no inh.	no inh.	no inh.	no inh.	no inh.
*Listeria monocytogenes*	18	no inh.	no inh.	no inh.	no inh.	no inh.	no inh.

Aerobic conditions	Diameter of growth inhibition zone (including well diameter), mm^b^						
		LA		LC		LA + LC	
	Positive control	Free	Encapsulated	Free	Encapsulated	Free	Encapsulated
*Escherichia coli*	no inh.	no inh.	no inh.	no inh.	no inh.	no inh.	no inh.
*Enterococcus faecalis*	no inh.	no inh.	no inh.	no inh.	no inh.	no inh.	no inh.
*Listeria monocytogenes*	no inh.	no inh.	no inh.	no inh.	no inh.	no inh.	no inh.

Abbreviations: LA, *Lactobacillus acidophilus*; LC, *Lacticaseibacillus casei*; no inh, no inhibition.

^a^ Incubation of anaerobic conditions.

^b^ Incubation of aerobic conditions.

## 4 CONCLUSION

The study aimed to increase the viability of probiotic bacteria, which are beneficial for human health, throughout the gastrointestinal process after food processing, storage, and consumption. Probiotic bacteria were encapsulated using the emulsion method with OLF, which is rich in phenolic compounds and has potential prebiotic properties. The optimum conditions for encapsulating LC and LA probiotic bacteria with maximum efficiency were determined using the central composite design–response surface methodology. The encapsulation efficiency of LC–OLF microcapsules was 93.66 ± 2.58%, while the encapsulation efficiency of LA–OLF microcapsules was 74.97 ± 1.34%, achieved under the optimum conditions. It was found that the prepared LC–OLF and LA–OLF microcapsules showed better resistance to storage conditions compared to free LC and LA probiotic bacteria. Additionally, the probiotic bacteria capsules showed increased resistance to simulated gastric fluid. Free bacteria could not survive gastric digestion and did not reach the intestinal environment. However, encapsulated LC and LA probiotic bacteria showed 39.59 ± 1.50% and 36.28 ± 0.01% survival, respectively, following in vitro gastrointestinal digestion. As a result, by encapsulating probiotic bacteria with oleaster flour, probiotic bacteria viability and survival rate were significantly increased under storage conditions and in vitro gastrointestinal digestion process.

## AUTHOR CONTRIBUTIONS

**Büşra Karkar:** Conceptualization (equal); investigation (equal); methodology (equal); writing – review and editing (equal). **Saliha Şahin:** Conceptualization (equal); investigation (equal); methodology (equal); writing – review and editing (equal). **Lütfiye Yılmaz‐Ersan:** Conceptualization (equal); investigation (equal); methodology (equal). **Bekir Akça:** Conceptualization (equal); methodology (equal). **Mesut Ertan Güneş:** Conceptualization (equal); methodology (equal). **Cüneyt Özakın:** Conceptualization (equal); methodology (equal).

## FUNDING INFORMATION

This research was financially supported by Bursa Uludağ University Scientific Research Projects Foundation (BAP) (Project No. FGA‐2021‐416).

## CONFLICT OF INTEREST STATEMENT

The authors have no conflict of interest in relation to this work.

## ETHICS STATEMENT

This study does not include experimental studies with animals or human participants that would require ethics committee approval.

## Data Availability

All relevant data are included in the manuscript and are also available from the corresponding author upon reasonable request.
